# Attenuation of Hyperoxic Lung Injury in Newborn Thioredoxin-1-Overexpressing Mice through the Suppression of Proinflammatory Cytokine mRNA Expression

**DOI:** 10.3390/biomedicines8030066

**Published:** 2020-03-20

**Authors:** Nobuhiko Nagano, Kosuke Tanaka, Junichi Ozawa, Takaaki Watanabe, Fuyu Miyake, Shun Matsumura, Kohei Osada, Kikumi Matsuoka, Masanori Tamura, Fumihiko Namba

**Affiliations:** 1 Department of Pediatrics, Saitama Medical Center, Saitama Medical University, Kawagoe, Saitama 350-8550, Japan; nagano.nobuhiko@nihon-u.ac.jp (N.N.); TANAKAK-PED@h.u-tokyo.ac.jp (K.T.); ozajun@saitama-med.ac.jp (J.O.); bluesky777brightsmile@gmail.com (T.W.); fuyumiyake@med.uoeh-u.ac.jp (F.M.); mshun1203@yahoo.co.jp (S.M.); ossa312@saitama-med.ac.jp (K.O.); kikumi@saitama-med.ac.jp (K.M.); mstamura@saitama-med.ac.jp (M.T.); 2 Department of Pediatrics and Child Health, Nihon University School of Medicine, Itabashi, Tokyo 173-8610, Japan

**Keywords:** antioxidant effects, hyperoxic lung injury, newborn mice, proinflammatory cytokine gene expression, thioredoxin-1

## Abstract

The role of thioredoxin-1 (TRX), a small redox-active protein with antioxidant effects, during hyperoxic lung injury in newborns remains undetermined. We investigated TRX impact on hyperoxic lung injury in newborn TRX transgenic (TRX-Tg) and wildtype (WT) mice exposed to 21% or 95% O_2_ for four days, after which some mice were allowed to recover in room air for up to 14 days. Lung morphology was assessed by hematoxylin/eosin and elastin staining, as well as immunostaining for macrophages. The gene expression levels of proinflammatory cytokines were evaluated using quantitative real-time polymerase chain reaction. During recovery from hyperoxia, TRX-Tg mice exhibited an improved mean linear intercept length and increased number of secondary septa in lungs compared with the WT mice. Neonatal hyperoxia enhanced the mRNA expression levels of proinflammatory cytokines in the lungs of both TRX-Tg and WT mice. However, interleukin-6, monocyte chemoattractant protein-1, and chemokine (C-X-C motif) ligand 2 mRNA expression levels were reduced in the lungs of TRX-Tg mice compared with the WT mice during recovery from hyperoxia. Furthermore, TRX-Tg mice exhibited reduced macrophage infiltration in lungs during recovery. These results suggest that in newborn mice TRX ameliorates hyperoxic lung injury during recovery likely through the suppression of proinflammatory cytokines.

## 1. Introduction

Bronchopulmonary dysplasia (BPD) is one of the most severe complications affecting premature infants [1]. Despite the remarkable improvements in neonatal-perinatal medicine, the overall incidence of BPD has not changed substantially. Recent studies have investigated the long-term consequences of BPD on the respiratory health of older children and adults. In extremely low-birth-weight survivors, who were born weighing less than 1000 g, lung function values at 8 and 12 years of age were lower than the reference values, especially in children with a history of severe BPD and in those exhibiting a bubbly/cystic appearance on chest X-rays [2]. Several pharmacological and nonpharmacological treatments have been proposed not only to support survival but also to minimize further lung injury and facilitate recovery in patients with BPD [3]. Except for protective ventilation strategies, optimal oxygen saturation targets, surfactant supplementation, and antenatal corticosteroid therapy, new therapies have not been efficient. Several alternative therapies using drugs such as inhaled nitric oxide and vitamin A have failed to consistently produce effective clinical outcomes, and most current therapies continue to be supportive [4,5]. Current studies are evaluating multiple therapies including caffeine, furosemide, systemic and inhaled corticosteroids, inositol, and cell therapy to characterize their efficacy and safety profiles [6].

Many novel therapeutic or preventive strategies to further improve outcomes and/or reduce the risk of BPD have to be investigated in animal models. Excessive oxidative stress due to exposure to high O_2_ concentrations has been reported to be a risk factor for the development of BPD [7]. Therefore, newborn mice exposed to high O_2_ concentrations have been commonly used as an animal model of BPD. This BPD model exhibits persistent alveolar and pulmonary vascular simplification resulting from delayed alveolar development; this histological finding is in accordance with the pathological changes observed in humans with BPD [8,9]. Furthermore, cardiac magnetic resonance imaging and echocardiography studies have demonstrated that hyperoxia-exposed mice exhibit signs of pulmonary hypertension and right ventricular dysfunction [10].

Thioredoxin-1 (TRX) is a ubiquitously expressed, small (12 kDa) endogenous antioxidant enzyme with a redox-active disulfide/dithiol within the conserved Cys-Gly-Pro-Cys sequence. TRX protects cells against oxidative stress by scavenging reactive oxygen species [11,12] while concurrently modulating chemotaxis and suppressing leukocytic infiltration to the sites of inflammation [13]. TRX transgenic (TRX-Tg) mice, in which the human TRX gene is systemically overexpressed under the control of the β-actin promoter, and mice in which recombinant human TRX is systemically administrated are resistant to injury in various models of human diseases including viral pneumonia [14], acute lung injury [15,16], pancreatitis [17,18], myocarditis [19], chronic obstructive pulmonary disease (COPD) [20,21], indomethacin-induced gastric injury [22], and lipopolysaccharide-induced preterm delivery [23]. The systemic overexpression of human TRX protects against hyperoxia-induced apoptosis in the cells of alveolar walls [24] and ameliorates significant protein leakage into the lungs, alveolar damage, focal alveolar hemorrhage, and hyaline membrane deposits by histological evaluation of the lungs, leading to significant reduction in mortality after exposure to hyperoxia in adult mice [25]. However, the effects of TRX overexpression in neonatal hyperoxia-induced lung injury, whose pathophysiology is very different than that of adult hyperoxia-induced lung injury [26], have not been determined. Therefore, the objective of the present study was to investigate the role of TRX in a mouse model of BPD by exposing newborn mice to hyperoxia, with the ultimate aim to determine the efficacy of recombinant human TRX as a novel therapeutic agent for the prevention and treatment of BPD in premature infants in future studies.

## 2. Materials and Methods

### 2.1. Animals

All protocols and procedures were approved by the Animal Care and Use Committee of Saitama Medical University (permit no. 1716, 13 November 2015). TRX-Tg mice were originally created by Takagi et al. [27]. Human TRX cDNA was inserted between the β-actin promoter and the β-actin terminator. The transgene was cut out of the plasmid by VspI and XbaI digestion, purified, and used to generate transgenic mice. The pronuclei of fertilized eggs from hyperovulated C57 BL/6 were micro-injectioned with this DNA construct. TRX-Tg mice were repeatedly backcrossed with C57 BL/6 mice for at least 12 generations and maintained in the animal facility at Saitama Medical Center, Saitama Medical University. Heterozygous TRX-Tg male and wildtype (WT) female mice were mated to produce either TRX heterozygous or WT littermates. Genotyping was performed using polymerase chain reaction (PCR) analysis of tail biopsies.

### 2.2. Neonatal Hyperoxic Exposure and Recovery

Newborn pups (< 12 h old) were randomly assigned to the normoxia (normal room air) or hyperoxia (95% O_2_) condition. Exposure to hyperoxia was performed for 96 h in a chamber (BioSpherix, Redfield, NY, USA) that allowed for the continuous monitoring and regulation of O_2_ and CO_2_. The dams were switched between hyperoxia and normoxia every 24 h. The inside of the chamber was maintained at atmospheric pressure, and the mice were exposed to a 12 h light/dark cycle. After hyperoxic exposure, some mice were allowed to recover in normoxia until they were 14 days old.

### 2.3. Lung Tissue Collection

Mice were anesthetized with intraperitoneal injection of pentobarbital (50 mg/kg) after determining the body weight. The pulmonary artery was perfused with phosphate-buffered saline via the right ventricle, and the right lung was excised and snap-frozen in liquid nitrogen for RNA and protein analyses. The left lung was inflated through the trachea with 10% neutral-buffered formalin (Sigma-Aldrich, St. Louis, MO, USA) at 25 cm gravity pressure and allowed to fix in situ for 1 min; the trachea was tied, and the left lung was then removed and fixed further overnight at 4 °C. The left lung tissue was paraffin-embedded, and 5-µm-thick sections were mounted onto glass slides for further analyses.

### 2.4. Lung Histology and Morphometry

Computer-aided morphometric analysis was performed using ImageJ software version 1.49 (NIH, Bethesda, MD, USA) to determine distal airspace maturation, and mean linear intercept length (Lm) and the number of secondary septa were determined.

Paraffin-embedded lung tissue sections were stained with hematoxylin and eosin. Lm, defined as the mean length of line segments on random test lines spanning the airspace between intersections of the line with the alveolar surface, was obtained using light microscopy by dividing the total length of a line drawn across the lung section by the total number of intercepts, until the number of intercepts reached 50 for each field [28]. Lm was assessed in six nonoverlapping fields of lung parenchyma in one tissue section per animal, and six animals per condition at each time point were examined.

Elastin staining was assessed using an elastic stain kit (Abcam, Cambridge, MA, USA) according to the manufacturer’s instructions. The number of secondary septa, where elastin was detected, was manually counted in six nonoverlapping fields of lung parenchyma in one tissue section per animal, and six animals per condition at each time point were examined.

### 2.5. RNA Extraction and Quantitative Real-Time PCR Analysis

Total RNA was extracted from five lung tissue samples per group, as previously described [29]. RNA (500 ng) was reverse-transcribed (High-Capacity cDNA Reverse Transcription Kit; Applied Biosystems, Foster City, CA, USA), and cDNA was amplified via PCR using primers (Applied Biosystems) for interleukin-6 (*Il-6*) (Mm00446190_m1), heme oxygenase-1 (*Ho-1*) (Mm00516005_m1), monocyte chemotactic protein-1 (*Mcp-1*) (Mm00441242_m1), *Il-1β* (Mm00434228_m1), tumor necrosis factor (*Tnf*) (Mm00443258_m1), chemokine (C-X-C motif) ligand 1 (*Cxcl1*) (Mm04207460_m1), *Cxcl2* (Mm00436450_m1), and macrophage migration inhibitory factor (*Mif*) (Mm01611157_gH) with the TaqMan Universal PCR Master Mix (Applied Biosystems). All PCR reactions were performed using a 7500 Fast Real-Time PCR System (Applied Biosystems). Relative mRNA expression levels were determined using the comparative critical threshold method, and each sample was normalized to β-glucuronidase (Mm01197698_m1) (Applied Biosystems).

### 2.6. Immunohistochemistry

Paraffin-embedded tissue sections were processed for immunofluorescence staining using the avidin/biotin-based peroxidase system (Vectastain ABC HRP Elite Kit; Vector Laboratories, Burlingame, CA, USA). Next, the sections were incubated with anti-F4/80 primary antibody (1:200; ab111101; Abcam) overnight at 4 °C, followed by incubation with a horseradish-peroxidase (HRP)-conjugated anti-mouse IgG secondary antibody (A10551; Invitrogen, Carlsbad, CA, USA) for 1 h at room temperature.

### 2.7. Western Blotting

Western blot analyses were performed to evaluate MIF protein levels. The blotted membranes were incubated with a primary anti-MIF (1:143; 524001; BioLegend, San Diego, CA, USA) or anti-β-actin (1:5000; GTX26276; GeneTex, Irvine, CA, USA) antibody, followed by HRP-labeled secondary antibodies. Chemiluminescence was detected using the Amersham ECL Prime Western blotting detection reagent (GE Healthcare, Little Chalfont, UK) with a digital imaging system (Bio-Rad ChemiDoc XRS+; Bio-Rad Laboratories, Hercules, CA, USA).

### 2.8. Immunoprecipitation

Lung 12,000× *g* supernatants from the hyperoxia-exposed WT or TRX-Tg mice were incubated with 2 μg hTRX antibody (ab133524; Abcam, Cambridge, UK) or nonimmune antibodies (normal rabbit IgG; Wako, Osaka, Japan) for 1 h at 4 °C. Next, 50 μL nProtein A Sepharose 4 Fast Flow suspension (GE Healthcare, Uppsala, Sweden) was added, and the mixture was incubated for 1 h at 4 °C. After centrifugation at 12,000× *g* for 20 s, the supernatants were removed and analyzed to detect MIF by Western blotting. Sample inputs were simultaneously loaded.

The following antibodies were used for Western blotting: Anti-MIF (1:286; 524001; BioLegend), anti-hTRX (1:2500; GTX108263; GeneTex), and anti-β-actin (1:5000; GTX26276; GeneTex).

### 2.9. Statistical Analysis

All values were expressed as means ± standard error of the mean. Comparisons between groups were performed using the statistical package JMP ver. 14 (SAS Institute, Cary, NC, USA) by one-way analysis of variance, followed by Tukey’s post hoc test for parametric data or the Kruskal–Wallis test followed by the Mann–Whitney *U* test for nonparametric data. *P*-values less than 0.05 were considered to indicate statistical significance.

## 3. Results

### 3.1. Neonatal Hyperoxia Does Not Negatively Affect Body Weight in TRX-Tg Mice

**Figure 1:**
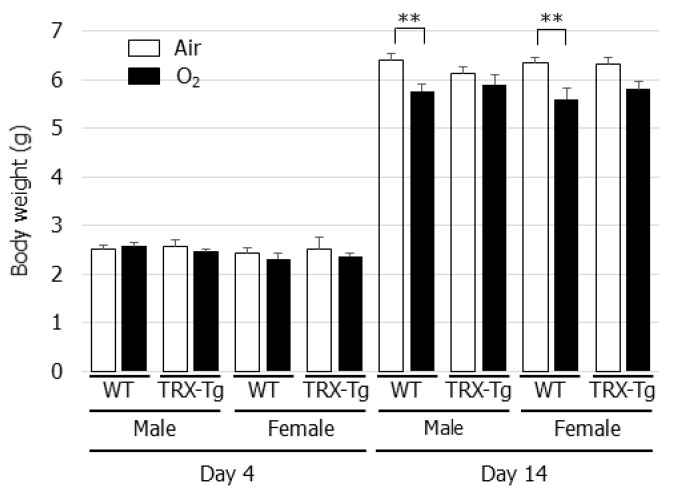
Changes in body weight after neonatal hyperoxic exposure. The changes in body weight were evaluated in 4 and 14-day-old WT and TRX-Tg mice following normoxic or hyperoxic exposure (*n* = 6 per group). Data are shown as means ± SEM. Comparisons between groups were performed using one-way ANOVA followed by Tukey’s test. ** *p* < 0.01. WT: wildtype; TRX: Thioredoxin-1; Tg: Transgenic; Air: Normoxia; O_2_: Hyperoxia; SEM: Standard error of the mean; ANOV: Analysis of variance.

The effect of neonatal hyperoxia on body weight was assessed in the WT and TRX-Tg mice. On day 4 after neonatal hyperoxia, no significant decrease in body weight was observed in the WT or the TRX-Tg mice compared with their normoxic counterparts. However, neonatal hyperoxia led to a reduction in body weight in both the male and female WT mice on day 14 during recovery from hyperoxia, whereas no change in body weight was observed in the TRX-Tg mice on day 14 following neonatal hyperoxia (Figure 1).

### 3.2. Alveolar Development Is Impaired After Neonatal Hyperoxia in Both WT and TRX-Tg Mice

**Figure 2:**
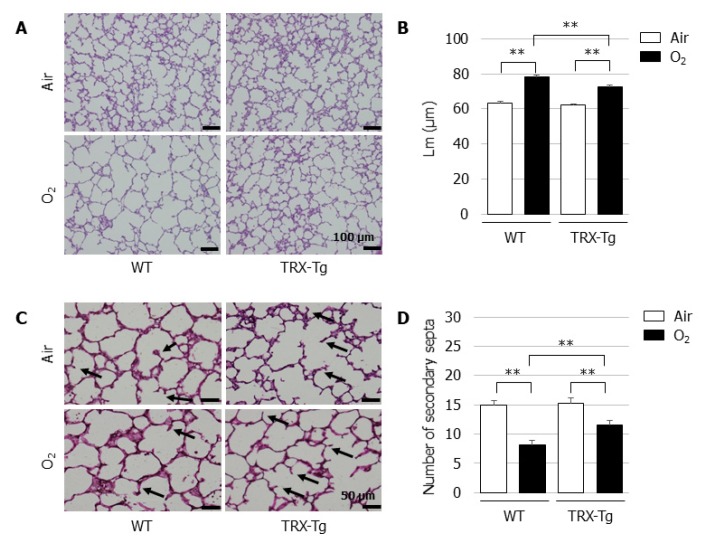
Alveolar development on day four after neonatal hyperoxic exposure. (**A**) H&E-stained histological sections. Scale bar = 100 µm (magnification × 100). (**B**) Lm (*n* = 6 per group). Lm was assessed in six nonoverlapping fields of lung parenchyma in one tissue section per animal. Animals were exposed to air (open bars) or O_2_ (filled bars). (**C**) Elastin-stained histological sections. The black arrows indicate elastin-positive secondary septa. Scale bar = 50 µm (magnification × 200). (**D**) Number of secondary septa (*n* = 6 per group). The number of secondary septa was assessed in six nonoverlapping fields of lung parenchyma in one tissue section per animal. Animals were exposed to air (open bars) or O_2_ (filled bars). Data are shown as the mean ± SEM. Comparisons between groups were performed using one-way ANOVA followed by Tukey’s test. ** *p* < 0.01. Air: Normoxia; O_2_: Hyperoxia; WT: Wildtype; TRX: Thioredoxin-1; Tg: Transgenic; Lm: Mean linear intercept length; H&E: Hematoxylin and eosin; SEM: Standard error of the mean; ANOVA: Analysis of variance.

In normal room air, the mice developed well-organized terminal airways. In contrast, the exposure of newborn mice to hyperoxia for 96 h led to the impairment of alveolar development, resulting in alveolar expansion and simplification (Figure 2A). Compared with the normoxic control lungs, a longer Lm was observed after neonatal hyperoxic exposure in the lungs of both the WT and TRX-Tg mice on day four (Figure 2B). Neonatal hyperoxia also led to a reduction in the number of secondary septa in the lungs of both the WT and TRX-Tg mice (Figure 2C,D).

### 3.3. Impaired Alveolar Development Is Mitigated in TRX-Tg Mouse Lungs during Recovery from Hyperoxia

**Figure 3:**
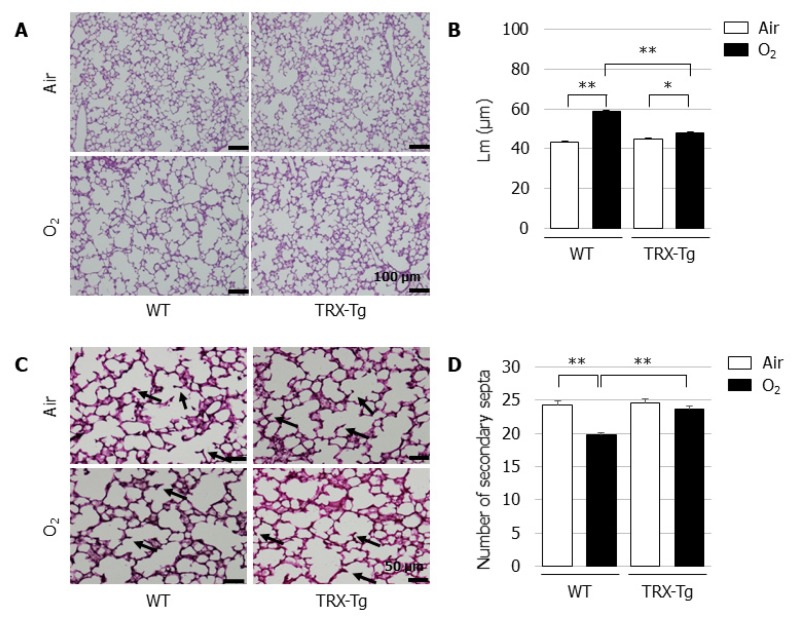
Alveolar development during recovery from neonatal hyperoxic exposure on day 14. (**A**) H&E-stained histological sections. Scale bar = 100 µm (magnification ×100). (**B**) Lm (*n* = 6, per group). Lm was assessed in six nonoverlapping fields of lung parenchyma in one tissue section per animal. Animals were exposed to air (open bars) or O_2_ (filled bars). (**C**) Elastin-stained histological sections. The black arrows indicate elastin-positive secondary septa. Scale bar = 50 µm (magnification × 200). (**D**) Number of secondary septa (*n* = 6 per group). The number of secondary septa was assessed in six nonoverlapping fields of lung parenchyma in one tissue section per animal. Animals were exposed to air (open bars) or O_2_ (filled bars). Data are shown as means ± SEM. Comparisons between groups were performed using one-way ANOVA followed by Tukey’s test. * *p* < 0.05; ** *p* < 0.01. Air: Normoxia; O_2_: Hyperoxia; WT: Wildtype; TRX: Thioredoxin-1; Tg: Transgenic; Lm: Mean linear intercept length; H&E: Hematoxylin and eosin; SEM: Standard error of the mean; ANOVA: Analysis of variance.

On day 14, the Lm was significantly longer and the number of secondary septa were significantly lower in the neonatal WT mice that were exposed to hyperoxia for four days followed by recovery in normal room air for ten days, compared with their normoxic counterparts. In contrast, despite the impaired alveolarization after neonatal hyperoxia on day 4, the TRX-Tg mice had a significantly improved Lm (Figure 3B) and exhibited an increase in the number of secondary septa (Figure 3D) after recovery in normal room air on day 14 compared with the WT mice that were exposed to the identical conditions.

### 3.4. Hyperoxia-Induced Increases in Proinflammatory Cytokine and Chemokine mRNA Expression Levels Disappeared During Recovery from Hyperoxia in TRX-Tg Mouse Lungs

**Figure 4:**
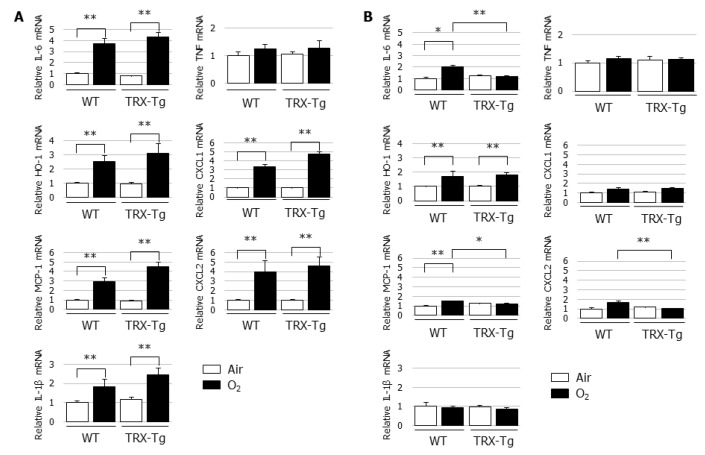
Changes in mRNA expression levels of *Il-6*, *Ho-1*, *Mcp-1*, *Il-1β*, *Tnf*, *Cxcl1*, and *Cxcl2* in lungs. Quantitative RT-PCR was performed on (**A**) day 4 and (**B**) day 14 (*n* = 6 per group). Animals were exposed to air (open bars) or O_2_ (filled bars). Data are shown as means ± SEM. Comparison between groups were performed using one-way ANOVA followed by Tukey’s test. * *p* < 0.05; ** *p* < 0.01. IL: Interleukin; HO: Heme oxygenase; MCP-1: Monocyte chemotactic protein-1; TNF: Tumor necrosis factor; CXCL: Chemokine (C-X-C motif) ligand; Air: Normoxia; O_2_: Hyperoxia; WT: Wildtype; TRX: Thioredoxin-1; Tg: Transgenic; SEM: Standard error of the mean; ANOVA: Analysis of variance.

TRX was reported to exert chemotaxis-modulating functions and to suppress leukocytic infiltration to sites of inflammation [20]. Therefore, the lung mRNA expression levels of proinflammatory cytokines and chemokines, including *Il-6*, *Mcp-1*, *Il-1β*, *Tnf*, *Cxcl1*, and *Cxcl2*, were determined using quantitative real-time PCR. Compared with the normoxic controls, *Il-6*, *Mcp-1*, *Il-1β*, *Cxcl1*, and *Cxcl2* mRNA expression levels were significantly increased in the lungs of both the WT and TRX-Tg mice after hyperoxic exposure on day four (Figure 4A). However, after a 10-day recovery in normal room air, the mRNA expression levels of *Il-6*, *Mcp-1*, and *Cxcl2* in the lungs of TRX-Tg mice exposed to neonatal hyperoxia returned to basal levels and were significantly lower than those in the hyperoxia-exposed WT mice (Figure 4B).

### 3.5. Macrophage Infiltration Is Reduced in the Lungs of TRX-Tg Mice after Neonatal Hyperoxia

**Figure 5:**
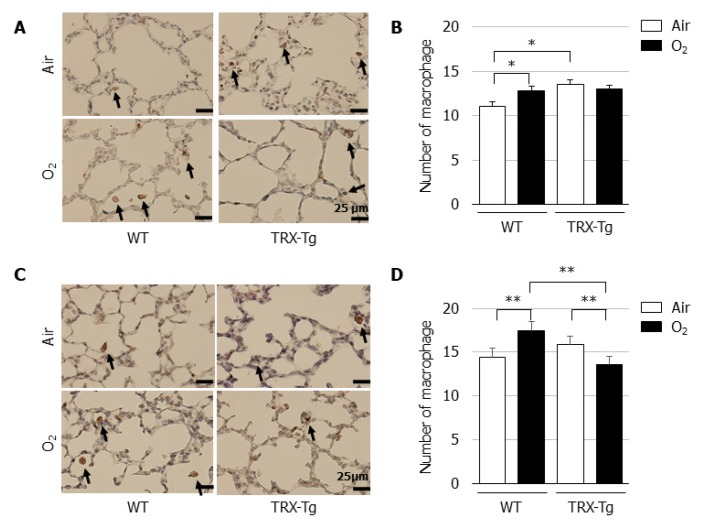
Immunohistochemistry for macrophages. Immunohistochemistry using the macrophage marker F4/80 was performed on lung sections on (**A**) day 4 and (**C**) day 14. The black arrows indicate F4/80-positive macrophages. Scale bar = 25 μm (magnification × 400). Number of macrophages on (**B**) day 4 and (**D**) day 14 (*n* = 6 per group). The number of macrophages was assessed in six nonoverlapping fields of lung parenchyma in one tissue section per animal. Animals were exposed to air (open bars) or O_2_ (filled bars). Data are shown as means ± SEM. Comparisons between groups were performed using one-way ANOVA followed by Tukey’s test. * *p* < 0.05; ** *p* < 0.01. Air: Normoxia; O_2_: Hyperoxia; WT: Wildtype; TRX: Thioredoxin-1; Tg: Transgenic; SEM: Standard error of the mean; ANOVA: Analysis of variance.

Based on the observed suppression of macrophage-related cytokine mRNA expression levels in the lung of TRX-Tg mice, we assessed the extent of macrophage infiltration in the lungs by immunohistochemistry using the macrophage marker F4/80. Compared with their normoxic counterparts, an increase in the number of macrophages was observed in the lungs of WT mice after hyperoxic exposure on day four and during recovery from hyperoxia on day 14. In contrast, in the lungs of TRX-Tg mice, although the higher basal macrophage numbers were observed under normoxic conditions, the hyperoxia-induced macrophage infiltration was inhibited on day 4 and the number of macrophages was decreased during recovery from hyperoxia on day 14 compared with the lungs of WT mice (Figure 5D).

### 3.6. Lung Mif mRNA and Protein Levels Are Not Different Between WT and TRX-Tg Mice after Hyperoxic Exposure

**Figure 6:**
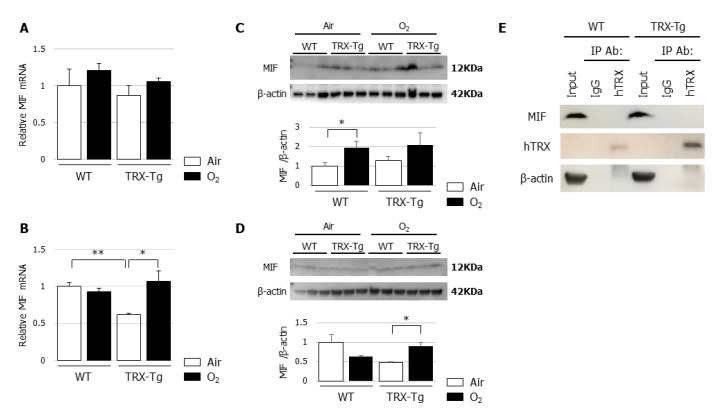
(**A**,**B**) *Mif* mRNA expression levels. Quantitative RT-PCR for *MIF* was performed on (**A**) day 4 and (**B**) day 14 (*n* = 6 per group). (**C**,**D**) MIF protein expression levels. Western blot analysis for MIF was performed on (**C**) day 4 and (**D**) day 14. Animals were exposed to air (open bars) or O_2_ (filled bars). Data are shown as means ± SEM. Comparisons between groups were performed using one-way ANOVA followed by Tukey’s test. * *p* < 0.05. Air: Normoxia; O_2_: Hyperoxia; MIF: Macrophage migration inhibitory factor; WT: Wildtype; TRX: Thioredoxin-1; Tg: Transgenic; SEM: Standard error of the mean; ANOVA: Analysis of variance. (**E**) Immunoprecipitation of TRX with MIF. Immunoprecipitation using normal IgG and anti-TRX antibody was analyzed by Western blot analysis. The antibodies for Western blotting were anti-MIF, anti-hTRX, and anti-β-actin. hTRX: Human thioredoxin; MIF: Macrophage migration inhibitory factor.

An in vitro study previously demonstrated the regulatory involvement of TRX in MIF internalization and signaling, such as the augmentation of TNF-α production, by the direct binding of TRX with MIF [30]. Therefore, we used quantitative RT-PCR and Western blotting to assess mRNA and protein expression levels of MIF and performed immunoprecipitation to assess the direct binding of TRX with MIF in our in vivo model. There were no differences in the mRNA and protein expression levels of MIF between the lungs of WT and TRX-Tg mice exposed to hyperoxia (Figure 6A–D), although the MIF mRNA and protein expression levels were increased in the lungs of TRX-Tg mice during recovery from hyperoxia on day 14 (Figure 6D). No direct binding was observed between TRX and MIF by immunoprecipitation followed by Western blotting (Figure 6E).

## 4. Discussion

In the present study, the newborn TRX-Tg mice tended to attenuate hyperoxia-induced lung injury compared with the newborn WT mice. During recovery from hyperoxia, the lungs of newborn TRX-Tg mice exhibited a shorter Lm and a greater number of secondary septa compared with the newborn WT mice, indicating that TRX overexpression mitigated the arrested alveolar development caused by neonatal hyperoxia. Furthermore, the mRNA expression levels of proinflammatory cytokines and chemokines, such as *Il-6*, *Mcp-1*, and *Cxcl2*, and the macrophage infiltration in the lungs of TRX-Tg mice exposed to neonatal hyperoxia were significantly suppressed on day 14 compared with the WT mice, suggesting that recovery from hyperoxic lung injury observed in the TRX-Tg mice might be due to the anti-inflammatory activity of TRX in the lungs of newborn mice. However, we did not observe an interaction between TRX and MIF in the lungs of TRX-Tg mice exposed to normoxia or hyperoxia.

Several animal models have shown the anti-inflammatory properties of TRX. We previously reported that recombinant human TRX attenuated the rate of lipopolysaccharide-induced preterm delivery in mice by preventing the elevation of proinflammatory cytokines including TNF-α, interferon-γ, MCP-1, and IL-6 in the maternal serum [23]. Using a mouse model of COPD exacerbation induced by cigarette smoke, Tanabe et al. demonstrated that TRX ameliorated neutrophilic inflammation by suppressing the release of granulocyte-macrophage colony-stimulating factor, thereby preventing the progression of emphysema, indicating the potential of TRX as a novel therapeutic agent that might counteract COPD exacerbation [31]. In a mouse model of irritant contact dermatitis induced by croton oil, topically applied recombinant human TRX significantly suppressed the inflammatory response by inhibiting the production of cytokines and chemokines, such as *Tnf-α*, *Il-1β*, *Il-6*, *Cxcl1,* and *Mcp-1*, in the skin tissues [32]. Furthermore, exogenous administration of recombinant human TRX significantly improved the survival rate and attenuated the histological changes in lungs in a mouse model of influenza pneumonia by diminishing the production of TNF-α and CXCL1 in the lungs observed in influenza-inoculated mice [33]. In agreement with these animal models, we observed the anti-inflammatory properties of TRX, including the suppression of mRNA expression levels of proinflammatory cytokines and chemokines, as well as the suppression of macrophage infiltration, in the hyperoxia-induced BPD model in newborn mice.

MIF, a proinflammatory cytokine, is released by T lymphocytes and can inhibit the random movement of macrophages [34]. In addition to T lymphocytes, MIF is secreted by a variety of other cells, including epithelial cells, endothelial cells, and macrophages [35]. MIF has been classified as a powerful cytokine, capable of inducing TNF-α, IL-1β, IL-6, and IL-8 and amplifying lipopolysaccharide-driven cytokine responses [36]. Son et al. demonstrated the specific affinity of TRX to MIF within cells, as well as culture supernatants of ATL-2 cells, which led to the MIF internalization into the ATL-2 cells; the authors also showed that MIF mediated the augmentation of TNF-α production from RAW264.7 macrophage cells [30]. In the current study, to examine the role of MIF in the observed differences between the WT and TRX-Tg mice during recovery from neonatal hyperoxia, we assessed the *Mif* mRNA and protein expression levels, direct binding of TRX with MIF, and *Tnf* gene expression levels in the lungs. In contrast to the previously reported in vitro findings, MIF and its downstream target TNF did not appear to be involved in the protective effects of TRX in the hyperoxic lung injury model used in the present study.

In clinical settings, extracellular concentrations of TRX were measured in various conditions characterized by oxidative stress and inflammation, including autoimmune diseases, ischemia-reperfusion injury, sepsis, viral infection, and acute lung injury [37,38,39,40]. Yu et al. reported that serum TRX might be a biomarker of cardioembolic stroke severity and that increased TRX levels might be a useful tool for predicting favorable prognosis in patients with acute ischemic stroke [41]. In patients with coronary artery disease accompanied with hyperhomocysteinemia, decreased serum TRX levels were closely correlated with the extent and severity of coronary artery disease [42]. TRX levels in human neonates, especially very premature babies, who often suffer from BPD, have not yet been investigated. To consider the utility of TRX as a biomarker or a novel therapeutic agent for BPD, the association of TRX levels in human samples, such as serum and tracheal aspirates, with the development and/or severity of BPD should be investigated. Furthermore, preclinical studies in animal models of BPD are warranted to assess the efficacy of treatment with recombinant human TRX.

## 5. Conclusions

Hyperoxic lung injury was attenuated in the newborn TRX-Tg mice compared with the newborn WT mice. The mechanism for the observed beneficial effect of TRX on lung development might occur through the suppression of proinflammatory cytokine gene expression after neonatal hyperoxic exposure, in the absence of MIF involvement.
